# Laser Fusion of Mouse Embryonic Cells and Intra-Embryonic Fusion of Blastomeres without Affecting the Embryo Integrity

**DOI:** 10.1371/journal.pone.0050029

**Published:** 2012-12-05

**Authors:** Alexander Krivokharchenko, Artashes Karmenyan, Oleg Sarkisov, Michael Bader, Arthur Chiou, Avetik Shakhbazyan

**Affiliations:** 1 Max-Delbrück Center for Molecular Medicine, Berlin-Buch, Germany; 2 Biophotonics and Molecular Imaging Research Center, National Yang-Ming University, Taipei, Taiwan; 3 N. N. Semenov Institute of Chemical Physics, Russian Academy of Sciences, Moscow, Russia; 4 Institute of Biophotonics, National Yang-Ming University, Taipei, Taiwan; 5 Institute of Theoretical and Experimental Biophysics, Russian Academy of Sciences, Pushchino, Moscow Region, Russia; Brigham and Women’s Hospital, United States of America

## Abstract

Manipulation with early mammalian embryos is the one of the most important approach to study preimplantation development. Artificial cell fusion is a research tool for various biotechnological experiments. However, the existing methods have various disadvantages, first of them impossibility to fuse selected cells within multicellular structures like mammalian preimplantation embryos. In our experiments we have successfully used high repetition rate picosecond near infrared laser beam for fusion of pairs of oocytes and oocytes with blastomeres. Fused cells looked morphologically normal and keep their ability for further divisions *in vitro*. We also fused two or three blastomeres inside four-cell mouse embryos. The presence of one, two or three nuclei in different blastomeres of the same early preimplantation mouse embryo was confirmed under UV-light after staining of DNA with the vital dye Hoechst-33342. The most of established embryos demonstrated high viability and developed *in vitro* to the blastocyst stage. We demonstrated for the first time the use of laser beam for the fusion of various embryonic cells of different size and of two or three blastomeres inside of four-cell mouse embryos without affecting the embryo’s integrity and viability. These embryos with blastomeres of various ploidy maybe unique model for numerous purposes. Thus, we propose laser optical manipulation as a new tool for investigation of fundamental mechanisms of mammalian development.

## Introduction

Traditional microsurgical manipulation methods to study the early development of embryos rely on mechanical tools which inevitably cause traumas. It is extremely difficult, if not impossible, to have a mechanical effect on any part of the embryo or its intracellular structures without damaging the intactness of the embryo. However, the use of laser beams as nanosurgical tools opens new possibilities in experimental embryology. Affecting cellular and subcellular structures of embryonic cells without breaking the intactness of the embryo itself can lead to the understanding of the function of individual components or structures in the process of development. Corresponding data were already published for Drosophila and zebrafish embryos [1]. First results about functional enucleation of mammalian oocytes by laser irradiation of the metaphase plate were obtained in our experiments in mice [2] and repeated later on in pigs [3]. Furthermore, we showed that is possible to perform the entire mammalian cloning procedure at all stages of nuclear transplantation only with the use of laser [4].

Artificial cell fusion has potential as a research tool for various biotechnological experiments with mammalian gametes and embryos. Previous by blastomere membrane fusion was realized by polyethylene glycol [5], [6], inactivated Sendai virus [7], or electric field [8], [9].

Currently, electrofusion has become the most popular method for embryonic cell fusion due to its easy use and high efficiency. Electrofusion of blastomeres of two-cell embryos has been applied for tetraploid embryo generation in a number of mammalian species such as mice [9], [10], rabbit [11], pig [12], cattle [13], and rats [14]. However, electrofusion has some disadvantages. First of them is the impossibility to fuse selected cells within multicellular structures like preimplantation embryos. Moreover, detrimental effects of the electric field were recently demonstrated in oocytes, zygotes, and two-cell mouse and rat embryos [15].

Laser technology has been successfully employed for fusion of various cells for many years [16], [17]. In these early works cell fusion was performed with the UV-lasers, while UV irradiation is destructive for bio-systems.

We have reported, for the first time, the generation of viable tetraploid mouse blastocysts after laser fusion of two blastomeres of two-cell stage embryos using much less damaging IR picosecond laser [2]. Later, the IR laser mediated fusion of large blastomeres of parthenogenetic two-cell porcine embryos was demonstrated [18]. We also reported, for the first time, successful laser fusion of mouse oocytes with somatic cumulus cells [4] where we used the laser with the same parameters as laid down by [2].

The aim of this work was to employ laser beams for the fusion of various embryonic cells of different size and of two or three blastomeres inside four-cell mouse embryos without affecting the embryo integrity and viability.

## Results

### Laser Fusion of Two Oocytes

**Figure 1:**
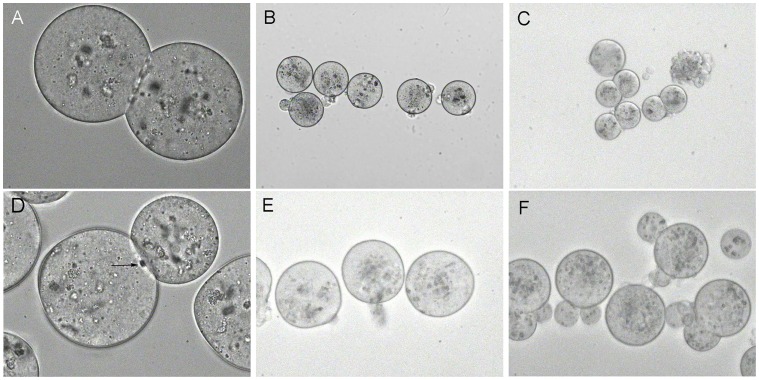
Oocyte-oocyte fusion and oocyte-blastomere fusion. (A) Two aggregated oocytes before fusion. (B) Fused oocytes, 60 min after laser treatment. (C) Development of fused oocytes after parthenogenetic activation and in vitro cultivation. (D) Laser irradiation of contact site between aggregated oocyte and blastomere (arrow show point of laser beams area of fusion). (E) Fused cells, 60 min after laser treatment. (F) Asymmetric cleavage of fused cells.

As a model for laser fusion of large embryonic cells freshly ovulated MII stage mouse oocytes were used. After removing of zona pellucida oocytes were aggregated in pairs in medium with lectin and the site of tight contact between the cells was treated by a laser beam (Figure 1A). Fused oocytes were selected (Figure 1B), activated by strontium and cultured *in vitro*. About half of pairs of aggregated oocytes fused –46.7% (21/45) and all of the ten cultured fused oocytes underwent normal cleavage (Figure 1C).

### Laser Fusion of Oocytes with Blastomeres

For laser fusion of large embryonic cells of different origin and size we used ovulated oocytes and single blastomeres from two-cell stage embryos. Oocytes and blastomeres were removed from zona pellucida and aggregated by lectin before laser treatment. The site of the tight cell contact was treated with a laser beam (Figure 1D). Fused pairs (21.2%–7/33) were selected (Figure 1E) and activated by strontium. Fused cells survived the procedures and most of them 85.7% (6/7) underwent cleavage after in vitro cultivation, however, cell divisions were asymmetric (Figure 1F).

### Intra-embryonic Laser Fusion of Blastomeres

In an other set of experiments, we used laser beams for the fusion of two or three blastomeres inside four-cell mouse embryos without affecting the embryo integrity. The presence of one, two or three nuclei in different blastomeres of the same early preimplantation mouse embryo was confirmed under UV-light after staining of DNA with the vital dye Hoechst-33342. The viability of the treated embryos was estimated by in vitro cultivation till blastocyst stage. The ability of non-treated embryos in control groups to reach the blastocyst stage was about 80%.

**Figure 2:**
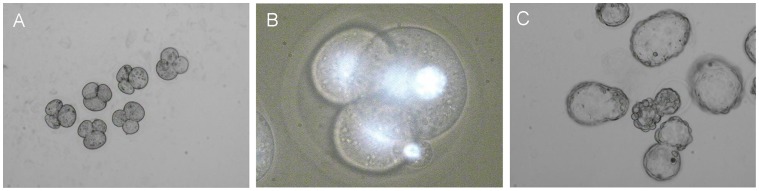
Fusion of two blastomeres inside 4-cell embryos. (A) 3-cell embryos as a result of fusion of two blastomeres inside 4-cell embryos. (B) Hoechst 33342 staining demonstrates two nuclei in one cytoplast. (C) Hatching blastocysts.

The Figure 2 (A, B) demonstrates the fusion of two blastomeres inside of 4-cell embryos. Fusion of blastomeres was proven in 61.5% (32/52) of treated embryos and 78.1% (25/32) of these embryos reached the blastocyst stage in culture. Some of the blastocysts even hatched from zona pellucida confirming their viability (Figure 2 C).

**Figure 3:**
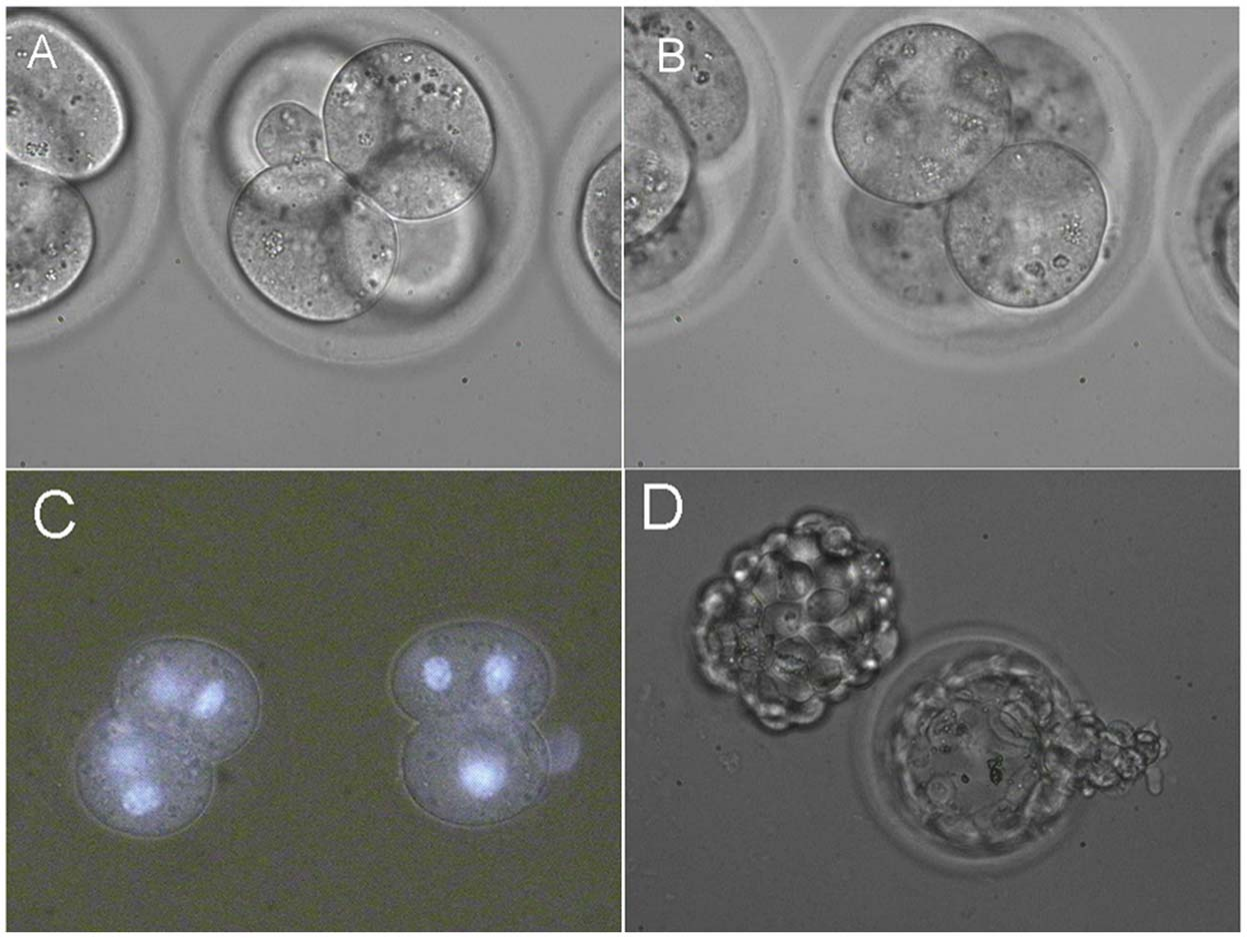
Fusion of two pairs of blastomeres inside 4-cell embryos. (A) Fusion of first pair of blastomeres. (B) Fusion of second pair of blastomeres. (C) Resulting 2-cell embryos. Hoechst 33342 staining confirms the presence of two nuclei in each blastomere. (D) In vitro development of 4-cell embryos with two fused pairs of blastomeres. Hatching and hatched blastocyst.

We also used laser beams for the fusion of two pairs of blastomeres inside 4-cell embryos. Pairs of blastomeres were fused sequentially (Figure 3A, B) with efficiency of 52.2% (12/23) and as a result we obtained 2-cell embryos with two nuclei in each blastomere (Figure 3C). After in vitro cultivation 90% (9/10) of them reached the blastocyst stage and some blastocysts hatched from zona pellucida (Figure 3D).

**Figure 4:**
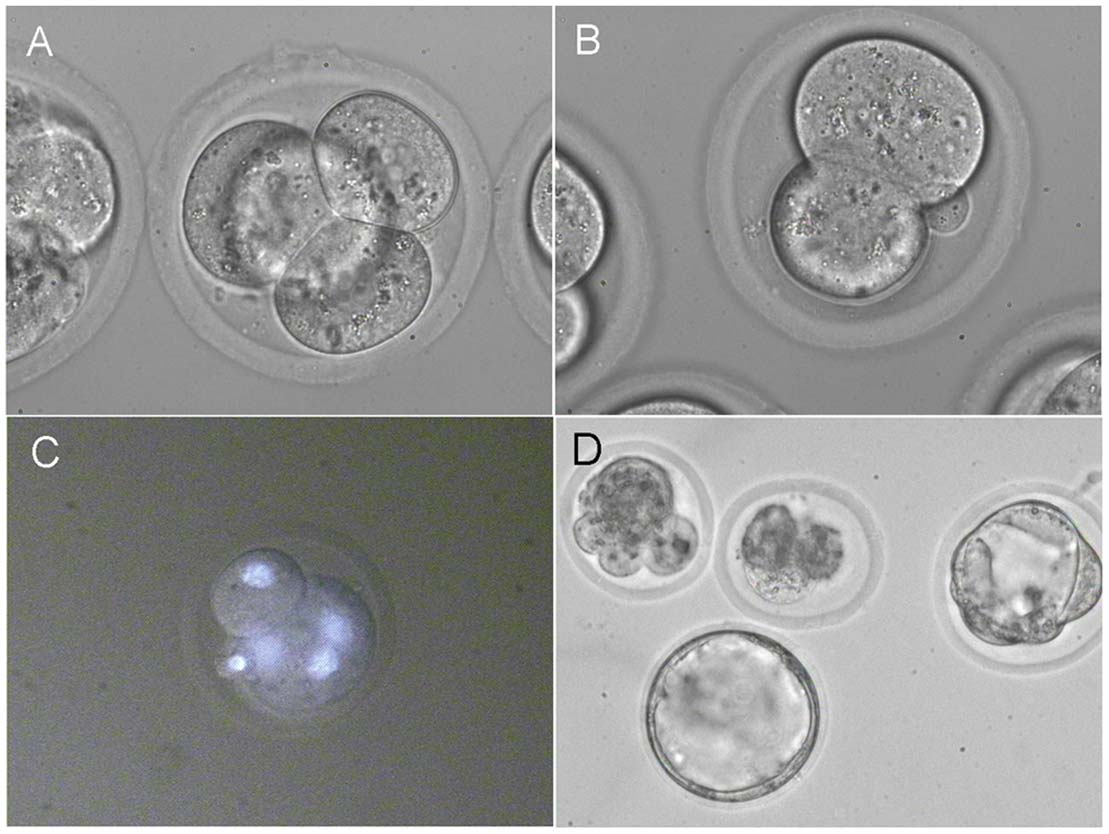
Fusion of three blastomeres inside 4-cell embryos. (A) 4-cell embryos with visible contacts between three blastomeres. (B) Embryo with one normal and one big blastomere from three fused blastomeres. (C) Hoechst 33342 staining confirms the presence of three nuclei in one cytoplast. (D) In vitro development of 4-cell embryos with three fused blastomeres. Middle stage and expanded blastocysts.

In the last set of experiments we fused three blastomeres inside 4-cell embryos. Successful fusion was shown in 44.4% (8/18) of the embryos (Figure 4 A–C) and half of the cultured embryos (3/6) developed in vitro till blastocyst stage (Figure 4D).

## Discussion

Unlike traditional methods of cell fusion such as chemical fusion, electrofusion and virus fusion laser does not affect whole cells and embryos but only defined points of membrane contact which may decrease detrimental effects and allows to fuse selected cells within multicellular structure like preimplantation mammalian embryos.

In our experiments we have demonstrated, for the first time, the fusion of different large cells of early mouse embryos, such as oocytes and blastomeres by laser beams without using any other tools and instruments. The laser fusion did not significantly affect cell viability.

These data allow us to propose that laser fusion may be successfully used instead of the most common fusion methods for many applications in various embryological experiments. Laser fusion of blastomeres at the two cell stage is a reliable technique for tetraploid embryo production and tetraploidy of all cells of laser created embryos was strongly proved by karyotyping as we reported [2]. Laser may be also used for the fusion donor karyoplasts with enucleated cytoplasts in somatic cell nuclear transfer (SCNT) as we demonstrated [4] and for increasing the oocyte volume after SCNT. Laser based methods may as be very efficient tools for novel developments in assisted reproductive technologies in humans e.g. for the introduction of large amounts of donor “young” cytoplasm in to oocytes from aged women and for the transfer of cytoplasm, germinal vesicle and pronuclei with the aim to eliminate the transmission of mitochondrial diseases in affected families.

We show here for the first time that laser beams can also be used for the fusion of two or three blastomeres inside four-cell mouse embryos without affecting embryo integrity and viability. Efficiency of fusion was quite high and varied from 61.5 to 44.4% depending on the number of fused blastomeres. Embryos with fused blastomeres demonstrated the ability to develop till blastocysts in vitro comparable to control non-treated embryos. It will be important to estimate total number of cells in blastocysts and the ratio of inner cell mass to trophectoderm cells.

In future experiments it will be interesting to use labeled blastomeres to study the fate of blastomeres with various ploidy and to investigate their ability to contribute to different cell lineages of blastocysts.

Chromosomal mosaicism in mammalian preimplantation embryos including phenomenon of polyploidy of some cells is well known. However, till now is not clear is it abnormalities or part of normal process of development. Laser fusion of selected blastomeres without destroying whole embryo can produce unique models for investigation the effect of different type of polyploidy of various blastomeres on embryo development.

Fusion by laser beam can be used to study the effect of changes in cell volume and nuclear-cytoplasmic ratio on blastomeres developmental ability.

Laser fusion of blastomeres inside intact embryo may be perspective for the creation of host embryos with blastomeres of various ploidy for the microinjection of pluripotent cells, such as embryonic stem cells and induced pluripotent stem cells, to study the distribution of polyploid host cells and pluripotent foreign cells in the resulting fetuses and offspring. From a practical point of view, it may be useful for obtaining offspring completely derived from embryonic stem cells or induced pluripotent stem cells.

In summary, laser fusion of defined cells of early preimplantation mammalian embryos and fusion selected cells inside embryos without affecting the embryo’s integrity should be an effective tool for numerous practical purposes and for the investigation of fundamental mechanisms of mammalian development.

## Experimental Procedures

### Ethics Statement

All experiments were performed according to the guidelines for the human use of laboratory animals by the Laboratory Animal Center of the National Yang-Ming University Taipei and all procedure were approved by Institutional Animal Care and Use Committee (IACUC) protocols (#981203 and #991256).

### Animals

The study was carried out in mice (CBA/Ca and C57BL/6) provided by the animal breeder National Applied Research Laboratories of National Laboratory Animal Center of Taiwan, Taipei. The mice were 1.5–2.5 months old. C57BL/6 female mice were superovulated by the standard method of intraperitoneal (i.p.) injection of 5 IU pregnant mare’s serum gonadotropin (PMSG) (Sigma) followed by an i.p. injection of 5 IU human chorionic gonadotropin (hCG) (Sigma) 48 hr later. After hCG injection, females of the first one group were used for obtaining oocytes, females of the other group were placed in cages with CBA/Ca males and examined the following morning for the presence of a vaginal plug (Day 1 of pregnancy). Mice were fed standard laboratory diet, provided water ad libitum.

### Oocytes and Embryos Recovery and Culture

The oocytes and zygotes were recovered from the excised oviducts (15–18 hr after the hCG injection) into M2 medium (Sigma) containing 0.1% (w/v) hyaluronidase (Sigma) to remove cumulus cells. In vivo produced 2-cell embryos were recovered from the excised oviducts 45–48 hrs after hCG. Then the ova were washed in M2 medium and used for manipulations. To obtain 4-cell stage embryos, 2-cell embryos were transferred to four-well culture dishes (Nunc) and cultured in M16 medium (Sigma) for 12–15 h under 5% CO_2_ in air at 37°C [14]. Previously, the culture medium was equilibrated with the gas phase and temperature in a CO_2_ incubator for 2–3 h. The same method of cultivation was used for estimation of embryo viability after laser treatment.

### Parthenogenetic Activation

Parthenogenetic activation of oocytes by strontium treatment was performed as described previously [19]. The oocytes were incubated for 30 min in Ca^2+^ and Mg^2+^- free M16 medium containing 2 mM Sr^2+^ at 37°C in a CO_2_ incubator.

### Manipulation of Ova

Oocytes and embryos were removed of their zona pellucida by treatment with acid Tyrode’s solution (Sigma) and then carefully washed in M2 medium. Oocytes and blastomeres were aggregated after 30 seconds incubation in M2 medium with 40 ug/ml phytohemagglutinin (lectin from Phaseolus vulgaris, Sigma). Nuclei staining was performed by 15 min incubating the embryos in M2 medium with 10 ug/ml specific DNA fluorescent dye, Hoechst 33342.

### Laser Setup

An “Olympus IX71” inverted microscope (Olympus, Japan) coupled with a mode-locked Ti:Sapphire laser (“Tsunami” Model 3950, Spectra-Physics, Mountain View, CA) pumped by a cw green laser (532 nm, 5 W, Millennia DPSSL, Spectra Physics, CA) working in a picosecond mode (∼2 psec FWHM) at a repetition rate of 80 MHz and a wavelength of 800 nm was used. The laser beam was expanded by a beam expander to completely fill the entrance pupil of the microscope objective and a programmable electronic shutter (UNI-Blitz) was used to control the exposure time in a wide range (from milliseconds to seconds). The average radiation power was varied within 0.08–0.8 W. In all experiments irradiation was performed with two different objectives (UPlanApo 20×, NA = 0.70 and UPlanFl 40× with NA = 0.75, Olympus, Japan). The average laser power was fixed at 0.4 W through out the experiments. A CCD camera (WAT-120N, Japan), connected to a PC via PCI-1411 (National Instruments), was used for continuous video digital recording with 1–2 frames per second.

### Laser Treatment

Optimal parameters of laser beam were selected and published early [2]. Fusion of cells were performed as described previously by laser beam irradiation of the middle region of the contact line between two cells without using any other tools and instruments. In the presented experiments the fusion of plasma membranes took place within 60–90 min after laser treatment. After 5–7 h irreversibility of fusion was estimated and all treated cells demonstrated fused state.
